# Disorders of the Oral Cavity in Parkinson's Disease and Parkinsonian Syndromes

**DOI:** 10.1155/2015/379482

**Published:** 2015-01-15

**Authors:** Yair Zlotnik, Yacov Balash, Amos D. Korczyn, Nir Giladi, Tanya Gurevich

**Affiliations:** ^1^Department of Neurology, Movement Disorders Unit, Tel Aviv Medical Center, 6423906 Tel-Aviv, Israel; ^2^Neurology Department, Soroka University Medical Center, 84101 Beer Sheva, Israel; ^3^Sackler School of Medicine, Tel-Aviv University, Tel-Aviv, Israel

## Abstract

Awareness of nonmotor symptoms of Parkinson's disease is growing during the last decade. Among these, oral cavity disorders are, although prevalent, often neglected by the patients, their caregivers, and physicians. Some of these disorders include increased prevalence of caries and periodontal disease, sialorrhea and drooling, xerostomia, orofacial pain, bruxism, and taste impairment. Though many of these disorders are not fully understood yet and relatively few controlled trials have been published regarding their treatment, physicians should be aware of the body of evidence that does exist on these topics. This paper reviews current knowledge regarding the epidemiology, pathophysiology, and treatment options of disorders of the oral cavity in Parkinson's disease patients.

## 1. Introduction

Although Parkinson's disease (PD) was generally considered a primary movement disorder for a long time, a majority, if not all, PD patients also suffer from nonmotor symptoms adding to the overall burden of the disease [1]. Awareness of nonmotor symptoms of Parkinsonism is growing during the last decade. Among these, oral cavity problems should not be neglected. These disorders include increased prevalence of gingivitis and dental diseases, diurnal and nocturnal sialorrhea and drooling, xerostomia, orofacial pain, the burning mouth syndrome, and bruxism [2].

The pathogenesis of these disturbances in PD may be multifactorial: some disorders occur due to general motor impairment and hypokinesia (dental and periodontal diseases due to difficulties in maintaining oral hygiene); others may be a manifestation of involuntary movements (facial dyskinesias/dystonia), due to medication (xerostomia), as a part of sensory dysfunction (taste impairment), or in relation to depressive symptoms (burning mouth syndrome, orofacial pain).

As we shall show, oral health remains a key component to the well-being of an individual diagnosed with PD [3]. The purpose of this paper is to review and summarize the current knowledge on this topic.

## 2. Dental Caries and Periodontal Disease

Poor oral health is common in PD patients [4–6]. Hanaoka and Kashihara evaluated the prevalence of periodontal disease by counting the numbers of caries and remaining teeth in consecutive 60- to 79-year-old patients with PD compared with two other patient groups: patients with mild neurological disease (without motor or cognitive impairment or diabetes mellitus, whose main complaint was tension-type headache) and persons with acute ischemic stroke. Patients with PD had fewer remaining teeth, more caries, and a higher prevalence of deep periodontal pockets. The frequency of patients with PD having untreated caries was high at Hoehn and Yahr stage II and above, particularly in those who scored low on mini-mental state examination [4]. Similar results were reported in other studies [7–9].

Patients with PD may experience difficulties in maintaining oral hygiene due to several factors: motor impairment, apathy, depression, and dementia [4]. As the disease progresses, it is therefore often up to a caregiver or family member to take the responsibility for oral hygiene because the patient with advanced PD can no longer perform adequate oral self-care [2].

Evidence is accumulating that systemic peripheral inflammation, such as gingivitis, may influence the progression of PD. Peripheral inflammation sparks off an exacerbation in the brain's ongoing damage in several neurodegenerative diseases, such as Alzheimer's disease (AD), multiple sclerosis, prion disease, stroke, and PD [10, 11].

These studies highlight the vulnerability of PD patients to dental caries, periodontal diseases, and teeth loss. They all suggest that clinicians and dentists should routinely check PD patients' oral health. Controlling oral cavity inflammatory disease should be an integral part of clinicians' efforts to prevent complications and improve the quality of lives of PD patients. Frequent dental visits are highly recommended and dental hygiene should be a part of the PD patient and caregiver education.

## 3. Sialorrhea and Drooling

Sialorrhea is defined as increased amount of accumulating saliva in the oral cavity, which may be caused by excessive production or by decreased clearance of saliva. In severe cases, this build-up of saliva may result in drooling, a common symptom in many neurological and otolaryngological disorders, but particularly in PD, where drooling may become a main concern with an important impact on patients' quality of life [12].

A recent study aimed to differentiate between diurnal and nocturnal drooling in PD and to evaluate the association between drooling severity and the severity of facial and oral motor deficits (such as reduced facial expressions, involuntary mouth opening, and swallowing complaints) [13]. Diurnal drooling—defined as dribbling of saliva while awake—was present in about 28% of PD patients. Nocturnal drooling (with or without diurnal drooling) was present in 58%. When compared to nondroolers, droolers were older and had more severe PD, longer disease duration, worse score on dysphagia and facial expression scales, and more severe involuntary mouth opening [13].

Diurnal drooling typically appeared later in the disease course and was associated with involuntary mouth opening and swallowing dysfunction. The study's data implied that drooling is generally not an early complaint and that it takes an average of 3 years to develop diurnal drooling after the patient had started to feel accumulation of saliva or noted a wet pillow when waking up in the morning [13].

In a meta-analysis based on 10 studies, Kalf et al. found that prevalence rates of drooling varied between 32 and 74%, depending on disease severity and definition of drooling. The mean prevalence in community-dwelling PD patients was 56% [13, 14].

Gender was found to be a significant factor in developing sialorrhea. Men were twice more likely to develop sialorrhea than women [15].

In general, sialorrhea may result from an excess production of saliva, difficulties in clearing saliva from the mouth (i.e., swallowing dysfunction), or both. The existing literature suggests that in PD, sialorrhea likely does not result from excess saliva production, but rather from a combination of infrequent or impaired swallowing (e.g., due to involvement of the dorsal motor nucleus of the vagus which might produce dysfunction of muscles controlling deglutition and esophageal motility) [12–16].

Several methods for assessment of sialorrhea in PD have been employed. Each has advantages and limitations and Chou et al.'s review is recommended for the reader who is interested in these tools [12]. All of these tools do not quantify the discomfort or social embarrassment related to sialorrhea.

Treatment options for sialorrhea in PD include pharmacological and nonpharmacological strategies. Among the pharmacological agents, anticholinergics have been studied as salivation is mediated primarily by parasympathetic innervation of the salivary glands.

Agents such as ipratropium bromide and glycopyrrolate are possibly helpful, at least for the short-term treatment of sialorrhea in PD. Botulinum toxins A and B are considered efficacious for the treatment of sialorrhea in PD. As far as safety is concerned, they have an acceptable risk with specialized monitoring due to the risk of transient swallowing difficulties including rarely severe dysphagia [1, 17].

Many nonpharmacological approaches to treating sialorrhea exist, but few of these have been applied to PD patients. These include a number of speech therapy interventions and devices, including oral motor therapy and orofacial regulation therapy. However these methods are probably more appropriate for neurologically impaired children with poor oral skills or hypotonic perioral musculature [12, 15, 18, 19]. A recent preliminary study by South et al. demonstrated that chewing gum may modify certain swallowing parameters and reduce drooling in PD patients [20].

Because dry mouth is a well-recognized side effect of radiation therapy for head and neck cancers, radiotherapy targeted at the parotid and submandibular glands has been proposed as a possible treatment for sialorrhea. Study cohorts included patients with sialorrhea secondary to various conditions; only some of them have PD, and it is not clear how the PD patients responded individually [12, 21].

Surgical treatment options for sialorrhea include neurectomy (sectioning of the chorda tympani nerves), salivary gland excision, salivary duct ligation, and salivary duct relocation. Duct ligation, duct relocation, and gland excision procedures can be done individually or in combination and have been performed mainly in neurologically impaired children. They are also more invasive than medications or botulinum toxin however, and potential complications include xerostomia and dental caries [12].

## 4. Xerostomia

Surprisingly, although sialorrhea is common in PD, dry mouth is a frequent complaint as well. In fact, xerostomia is one of the most common oral manifestations in individuals with PD and an instigator of both caries and periodontal disease. A survey conducted by Clifford and Finnerty showed that PD patients suffered from xerostomia at least twice as often as the general population [22]. It has been estimated that xerostomia affects approximately 55% of patients with PD [3].

Cersósimo et al. sought to determine whether hyposialorrhea is an early manifestation of PD. They measured basal and citric acid stimulated secretions of saliva in 20 patients with early stage (Hoehn-Yahr I-II) PD who had motor symptoms for less than one year and were on no medication and 11 age matched controls. Compared to controls, PD patients had significant reduction of both basal and stimulated salivary secretions. These findings confirm that hyposialorrhea is an early autonomic manifestation of PD [23].

Xerostomia could be drug induced, and indeed hundreds of drugs may be xerogenic. The drugs that most commonly induce dry mouth are the tricyclic antidepressants, antipsychotics (particularly clozapine), anticholinergics, beta-blockers, and antihistamines [24]. PD patients often suffer from nonmotor symptoms such as psychiatric and urinary disorders which may require treatment by xerogenic drugs.

Pharmacological options for alleviating xerostomia include cholinergic agonists such as pilocarpine HCl tablets (5 mg) or cevimeline HCl (available only in the US), which can stimulate the saliva production; however studies regarding these agents in PD are lacking [2, 25].

Recently, orally administered ubiquinol (CoQ10) 100 mg/day was shown to significantly improve salivary secretion (up to 80%) in dry mouth non-Parkinsonian patients [26].

Nonpharmacological strategies to alleviate xerostomia include sipping water frequently, utilizing sugar-free hard candies, frequently chewing sugar-free, xylitol-containing gum, or using various salivary substitutes [2].

## 5. Orofacial Pain and Burning Mouth Syndrome

The presence of pain and other abnormal sensations in PD is well documented. A survey evaluating the patients' perception of their most troublesome symptoms found that pain ranked high in all stages of disease [27, 28].

Parkinsonian pain does not frequently involve the head or face [29], yet oral pain syndromes have been reported as a non-motor off symptom, and as such may respond to dopaminergic therapy [27].

The burning mouth syndrome (BMS) is characterized by a painful, intraoral burning sensation that lacks physical or laboratory correlates. In the general population, postmenopausal women and the elderly are most often affected, with a prevalence ranging from 3.7 to 18% [30, 31]. BMS may be more common in PD with one study reporting a 24% prevalence [30, 32].

Psychological factors such as anxiety, depression, compulsive disorders, psychosocial stress, and cancer phobia may play a role in BMS [33]. High prevalence of depression and anxiety among PD patients may explain a higher prevalence of BMS in PD. It is still unknown whether treatment of depression can alleviate BMS.

While the pathophysiology is unclear, decreased dopamine levels and dopamine dysregulation were hypothesized to play a role [30, 34, 35].

Clinical trials have found that drug therapy with capsaicin, alpha-lipoic acid, clonazepam, and antidepressants may provide relief of oral burning or pain symptom. In addition, psychotherapy and behavioral feedback may also help alleviate the BMS symptoms [33].

Pramipexole has been reported as a therapy in primary BMS [36]. According to a case report, a single PD patient who developed BMS while receiving carbidopa/levodopa had resolution of the symptoms when the drug was replaced by pramipexole. This may support its use in the treatment of BMS in PD patients, but obviously this anecdotal finding should be confirmed by a pilot study, and if positive, by clinical trials.

## 6. Mastication Disorders

Masticatory functions seem to be impaired in PD in several ways. The jaw mobility and the speed of the jaw movements are reduced. Rigidity, reduced mobility of the tongue and jaw, and jaw tremor complicate the formation and the placement of the food bolus, the chewing process, and the swallowing. Mastication and orofacial functions are more impaired in moderate-advanced PD and increase with its progression [37].

As the maintenance of a reasonable number of healthy natural teeth is of paramount importance to obtain and secure masticatory efficiency, dental disease and decay should be prevented and treated in order to diminish and compensate for the impairment in the control of masticatory and tongue muscles [37].

## 7. Bruxism

Bruxism refers to an oral activity such as clenching, grinding, or bracing of teeth that can occur in sleep or awake state [38, 39]. Severe bruxism is implicated in causing tooth damage, temporomandibular disorders, swallowing and speech difficulties, headaches, and depression [40].

Bruxism has been reported in PD [41, 42] as well as in other Parkinsonian syndromes, such as MSA [43] and normal pressure hydrocephalus [38], and also in relation to levodopa treatment [44].

A recent study has shown a higher rhythmic masticatory muscle activity typical of sleep bruxism in REM sleep behavior disorder (RBD), which is known to be more prevalent in PD than in healthy subjects. This rhythmic masticatory muscle activity was found to be higher also in non-REM sleep in PD subjects compared to controls [45].

Botulinum toxin injections [42] and mechanical devices such as oral splints may be used for the management of bruxism. Other options include benzodiazepines, dopaminergic drugs, anticonvulsants, antidepressants, and sympatholytic drugs like clonidine, but level of evidence regarding these agents is low and further efficacy and safety assessments are needed before clinical recommendations can be made, particularly in PD [46].

## 8. Subjective Taste Impairment

Taste impairment includes reduced, altered, or sensitized sensation and has been reported in PD, but less frequently than the more prevalent olfactory impairment (9–27%) and with contradictory results [47–50].

Kashihara et al. reported that PD patients with smell impairment showed a higher frequency (25%) of taste impairment when compared with PD patients without smell impairment (9%). The frequency of taste impairment tended to increase with disease progress. Taste sense may be altered not only by central nervous system degeneration, but also by a variety of mental and physical conditions such as depression, reduced saliva secretion, poor oral hygiene, gastrointestinal diseases, zinc deficiency, medication, and smoking [47]. These factors also may explain, in part, the causes of taste impairment complaints, especially in PD patients without smell impairment.

Taste impairment has clinical relevance as it may lead to loss of appetite and subsequent malnutrition.

## 9. Dental Treatments

Dental hygienists should be aware of PD patients' vulnerability and special needs in order to implement strategies that may ensure a caring and effective treatment. It is preferable for dental hygienists to schedule the patient with PD 60–90 min after their medications have been taken, as medications tend to be most effective in that time period, rendering improved treatment conditions. Stress may exacerbate uncontrolled movements making any dental treatment more complicated. Further patient management strategies are reviewed by Debowes et al. [2].

Additionally, oral surgeons, dentists, and referring physicians should be aware of a potential severe interaction between deep brain stimulation (DBS) and diathermy. Several authors have reported severe neurological complications following diathermy use in DBS implanted patients [51, 52]; thus diathermy use is contraindicated in this population. Using electrocautery near an implantable neurostimulator may result in component failure or induced currents that may be harmful to the patient. In general, it is recommended to turn off the stimulator if this is medically appropriate, especially when some kind of electrocautery/electrosurgery is planned during the procedure, and to use only bipolar cautery.

## 10. Conclusions

Various disorders associated with the oral cavity may be present in PD patients, causing embarrassment, suffering, and physical damage.

Studies suggesting a link between systemic inflammation and progression of PD further highlight the importance of preventing oral cavity inflammatory insults, which could serve as the culprits for disease progression and complications. Other disorders may contribute to weight loss, depression, sleep disturbances, and pain, severely affecting PD patients' quality of life.

These disorders are frequently neglected by the patients, their caregivers, and their treating physicians.

Most of these disorders have not been fully addressed and further studies are needed in order to understand their pathophysiology and to offer optimal treatment.

Treating physicians and dentists should be aware of PD patients' special needs, and frequent dental visits should be recommended in order to prevent some of these complications.
